# Fourier analysis of circumpapillary retinal nerve fiber layer thickness in optical coherence tomography for differentiating myopia and glaucoma

**DOI:** 10.1038/s41598-020-67334-6

**Published:** 2020-06-29

**Authors:** Ming-Hung Hsieh, Yu-Fan Chang, Catherine Jui-Ling Liu, Yu-Chieh Ko

**Affiliations:** 1 Department of Ophthalmology, Taipei Veterans General Hospital, No. 201, Shih-Pai Rd., Sec. 2, Taipei, 11217 Taiwan; 2 Institute of Clinical Medicine, National Yang-Ming University, No. 155, Sec. 2, Linong St., Beitou Dist., Taipei, 11221 Taiwan; 3 Faculty of Medicine, National Yang-Ming University, No. 155, Sec. 2, Linong St., Beitou Dist., Taipei, 11221 Taiwan; 4 Department of Ophthalmology, Taipei City Hospital, No. 33, Sec. 2, Zhonghua Rd., Zhongzheng Dist., Taipei, 10065 Taiwan

**Keywords:** Retina, Computational models, Diagnostic markers

## Abstract

Differentiating glaucoma from myopic eye is a challenge to ophthalmologists. We try to develop a new discrete Fourier transform (DFT) model for analyzing optical coherence tomography data for the circumpapillary retinal nerve fiber layer (cpRNFL), and investigate DFT as a new diagnostic tool for glaucomatous myopic eyes. The thicknesses of 12 equidistant cpRNFL points were transformed into 6 signals in the frequency domain, ranging from 1 to 6 Hz. In all 232 eyes, generalized linear model showed that 1 Hz, 2 Hz, and 4 Hz were associated with glaucoma, high myopia, and the interaction between glaucoma and high myopia. The 3 Hz signal was associated with glaucoma and high myopia exclusively. A receiver operating characteristic curve analysis of the 3 Hz signals showed areas under the curves of 0.93 (95% CI 0.90–0.96) and 0.93 (95% CI 0.88–0.98), for diagnosing glaucoma in all subjects and in the highly myopic group, respectively. The DFT model is useful to differentiate glaucoma from non-glaucomatous change and showed potential as a diagnostic tool for glaucomatous myopic eyes.

## Introduction

Myopia is the most common ocular problem in the world. The prevalence of myopia in Asia is higher than that in Africa, Europe, or America.^1-4^ Globally, it is estimated that, between the present and the year 2050, the prevalence of myopia and high myopia will continue to grow.^5^

Glaucoma is one of the leading causes of blindness. It is characterized as progressive degeneration of retinal ganglion cells and thinning of the circumpapillary retinal nerve fiber layer (cpRNFL), which is the superficial layer of retina in the human eye. In recent years, optical coherence tomography (OCT) has become an important tool for detecting early anatomical changes in the cpRNFL. OCT is currently used for disease screening and progression assessments. However, in high myopic eyes, analyses of OCT images of the cpRNFL have resulted in high false-positive error rates, which makes the diagnosis of glaucoma complicated.^6,7^

Clinically, visual field examination, which reveals the patient’s optic nerve function, is the gold standard test to diagnose and evaluate the progression of the glaucoma. However, it takes about half an hour to perform the test in the both eyes of a patient with a whole-process accompanied technician. It was a huge burden for patients and medical system to treat glaucoma according to the visual field test. OCT investigates the structure of the optic nerve fiber layer in the eyes by non-invasive measurement, and the test takes only few minutes. In addition to the convenience, it detects the abnormality of structure changes which is more sensitive to early pathological changes than detecting the functional abnormality.

Myopia, axial elongation of the eyeball, is associated with the appearance of an optic nerve crescent, disc tilt, disc torsion, and thinning of the peripapillary RNFL in some sectors, in the absence of glaucomatous change.^8-10^ Those characteristic findings, which are usually associated with glaucoma, are found in high myopic eyes, due to the posterior wall of the eyeball is thinner, more irregular, and more curved. To the best of our knowledge, it is difficult to identify glaucomatous changes in the RNFL of high myopic eyes, and current methods are unreliable.

Discrete Fourier Transform (DFT) is a method for transforming one function into another, and it is widely employed in signal processing and related fields. The DFT method requires input of samples from an equidistant, discrete function, such as the thicknesses of the RNFL at points around a clock-hour map, acquired with OCT The purpose of this study was to develop a new model of DFT analysis of OCT images of the RNFL and investigate whether this approach could serve as a new diagnostic tool for evaluating glaucoma in myopic eyes.

## Results

We included 232 subjects (123 males), with a mean age of 49.6 years (16–85). Of the 232 eyes studied, high myopia was found in 27 (34%) of 80 control eyes and 79 (52%) of 152 glaucomatous eyes.

With DFT application, the OCT RNFL signals were transformed to amplitudes in different frequency domains, which ranged from 0 to 6 Hz (Table 1).

**Table 1:** Demographic and the transformed data of frequency domain in control and glaucoma group.

	Control N = 80		Glaucoma N = 152		*p*
	N (%)	Mean (SD)	N (%)	Mean (SD)	
Male	27 (34)		96 (63)		< 0.01
Age (year)		44.08 (13.90)		52.54 (14.69)	< 0.01
High myopia	27 (34)		79 (52)		0.01
Axial length (mm)		25.40 (1.77)		26.05 (1.79)	0.01
Vertical C/D ratio		0.56 (0.17)		0.80 (0.12)	< 0.01
MD^a^ (dB)		− 1.46 (1.82)		− 5.24 (3.28)	< 0.01
Clockhour of RNFL ($\upmu$m)^b^					
1		99.44 (21.76)		85.92 (20.92)	< 0.01
2		74.44 (14.45)		70.50 (12.49)	0.03
3 (nasal)		58.09 (11.23)		59.65 (11.65)	0.33
4		60.41 (11.22)		60.58 (11.49)	0.92
5		85.04 (17.91)		76.08 (18.76)	< 0.01
6 (inferior)		119.23 (27.61)	79.77 (25.46)	< 0.01	
7		150.81 (22.63)		77.98 (29.75)	< 0.01
8		89.93 (18.65)		60.41 (17.19)	< 0.01
9 (temporal)		65.36 (13.48)	55.96 (11.61)	< 0.01	
10		100.31 (21.54)		68.80 (19.23)	< 0.01
11		136.59 (21.36)		93.48 (31.08)	< 0.01
12 (superior)		103.14 (26.00)	85.92 (20.92)	< 0.01	
Frequency of DFT^c^					
0 Hz		1,142.78 (108.94)		886.25 (133.82)	< 0.01
1 Hz		117.53 (64.59)		82.64 (41.12)	< 0.01
2 Hz		181.39 (71.01)		108.52 (59.94)	< 0.01
3 Hz		114.80 (33.69)		48.41 (27.82)	< 0.01
4 Hz		68.59 (31.82)		45.94 (25.69)	< 0.01
5 Hz		34.45 (17.32)		31.46 (17.41)	0.22
6 Hz		49.08 (27.80)		35.21 (25.76)	< 0.01

^a^Mean deviation of visual field.

^b^Retinal nerve fiber layer.

^c^Discrete Fourier transform.

### Discrete Fourier transform in control group

In the control population, the mean amplitude was 1,142.8 ± 108.9 at a mean thickness, or zero frequency (0 Hz). The amplitudes were 117.5 ± 64.6 at 1 Hz, 181.4 ± 71.0 at 2 Hz, 114.8 ± 33.7 at 3 Hz, 68.6 ± 31.8 at 4 Hz, 34.5 ± 17.3 at 5 Hz, and 49.1 ± 27.8 at 6 Hz. The regression analyses showed that increases in amplitude at 1 Hz and 4 Hz and a decrease in amplitude at 2 Hz and 5 Hz were associated with longer axial lengths. Increases in amplitude at 2 Hz and decreases in amplitude at 1, 4, and 6 Hz were associated with aging. The amplitude at 3 Hz was not associated with axial length or age in control group (Table 2) and also in glaucoma group (Table 3).

**Table 2:** Linear regression analysis of the association between hertz in frequency domain and axial length, and the association between hertz in frequency domain and age in control group.

Frequency domain (Hz)	Axial length			Age		
	$\mathrm{B}$	95% CI	*p*	$\mathrm{B}$	95% CI	*p*
0	− 31.46	− 43.36 to − 19.56	< 0.01	− 0.91	− 0.84 to 2.67	0.30
1	9.49	1.56 to 17.42	0.02	− 1.45	− 2.45 to − 0.46	< 0.01
2	− 24.95	− 32.01 to − 17.88	< 0.01	1.42	0.32 to 2.53	0.01
3	− 2.36	− 6.61 to 1.89	0.27	− 0.30	− 0.85 to 0.24	0.27
4	7.22	3.52 to 10.92	< 0.01	− 1.15	− 1.59 to − 0.70	< 0.01
5	− 2.40	− 4.53 to − 0.26	0.03	0.22	− 0.06 to 0.50	0.12
6	− 0.82	− 4.35 to 2.71	0.65	− 0.60	− 1.03 to − 0.17	< 0.01

B, the coefficient of linear regression equation; CI, confidence interval.

**Table 3:** Linear regression analysis of the association between hertz in frequency domain and axial length, and the association between hertz in frequency domain and age in patients with glaucoma.

Frequency domain (Hz)	Axial length			Age		
	$\mathrm{B}$	95% CI	*p*	$\mathrm{B}$	95% CI	*p*
0	− 7.88	− 19.84 to 4.08	0.20	0.94	− 0.52 to 2.41	0.21
1	− 3.25	− 6.91 to 0.41	0.08	0.29	− 0.16 to 0.74	0.21
2	− 9.75	− 14.90 to − 4.60	< 0.01	0.66	0.01 to 1.31	0.05
3	− 1.25	− 3.74 to 1.24	0.32	0.11	− 0.20 to 0.42	0.48
4	− 1.04	− 3.34 to 1.27	0.38	− 0.11	− 0.39 to 0.18	0.45
5	0.65	− 0.92 to 2.21	0.42	− 0.11	− 0.30 to 0.08	0.25
6	− 1.05	− 3.36 to 1.26	0.37	− 0.08	− 0.36 to 0.20	0.57

$\mathrm{B}$, the coefficient of linear regression equation; CI: confidence interval.

### Discrete Fourier transform model

In all 232 eyes, a comparison between glaucoma and control groups showed that amplitudes were significantly lower in the glaucoma group at 0, 1, 2, 3, 4, and 6 Hz.

A generalized linear model adjusted for age showed that glaucoma had a clear effect on amplitude at 0 Hz. At 1 and 2 Hz frequencies, glaucoma, high myopia and the interaction between glaucoma and high myopia were associated with reduced amplitudes. At the 3 Hz and 6 Hz frequencies, glaucoma and high myopia were associated with reduced amplitude, respectively (Table 4). At 4 Hz frequency, glaucoma was associated with reduced amplitude, however, high myopia was associated with increased amplitude.

**Table 4:** The association and interaction of glaucoma and high myopia using hertz of frequency in RNFL discrete Fourier transformation presented by generalized linear model analysis.

	0 Hz	1 Hz	2 Hz	3 Hz	4 Hz	5 Hz	6 Hz
	*p*						
Glaucoma	< 0.01	< 0.01	< 0.01	< 0.01	< 0.01	0.54	0.04
High myopia	0.01	0.02	< 0.01	0.01	< 0.01	0.34	0.02
High Myopia with Glaucoma	0.02	< 0.01	< 0.01	0.65	< 0.01	0.07	0.87

All analysis was adjusted by age.

**Figure 1:**
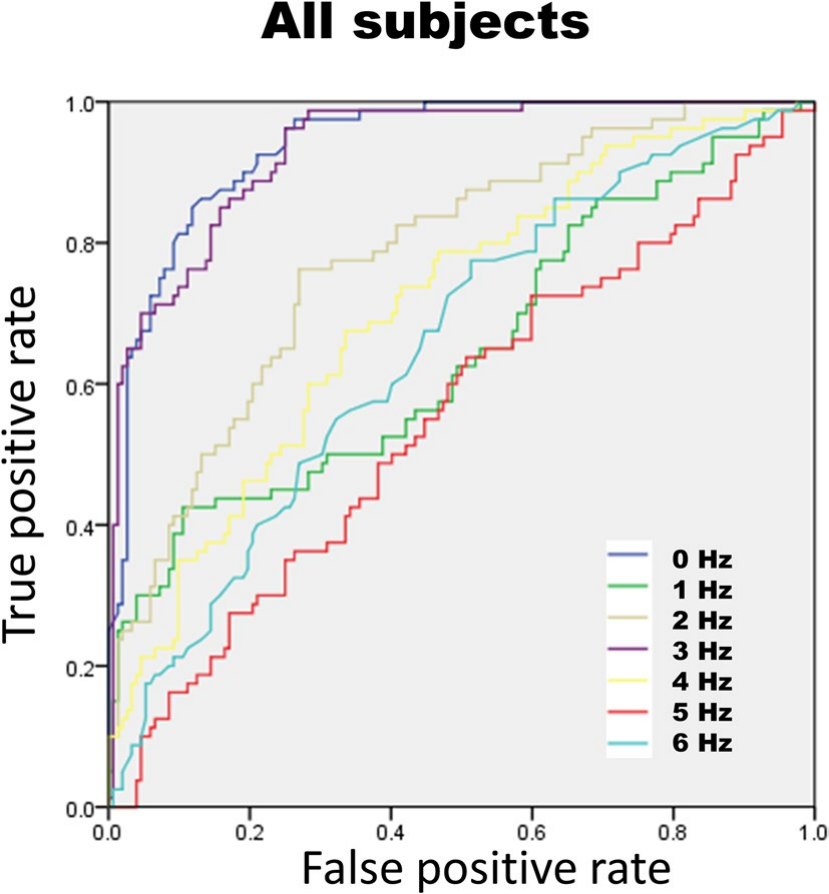
Receiver operating curve of amplitude 0–6 Hz frequency for all subjects. In all subjects, there was better area under curve in 0 Hz and 3 Hz (0.939 and 0.933) than other biomarkers. The 0 Hz, which is the mean of thickness, is the current biomarker to detect and diagnose the glaucoma. The image was created using SPSS software (ver. 17.0; SPSS Inc, Chicago, IL).

**Figure 2:**
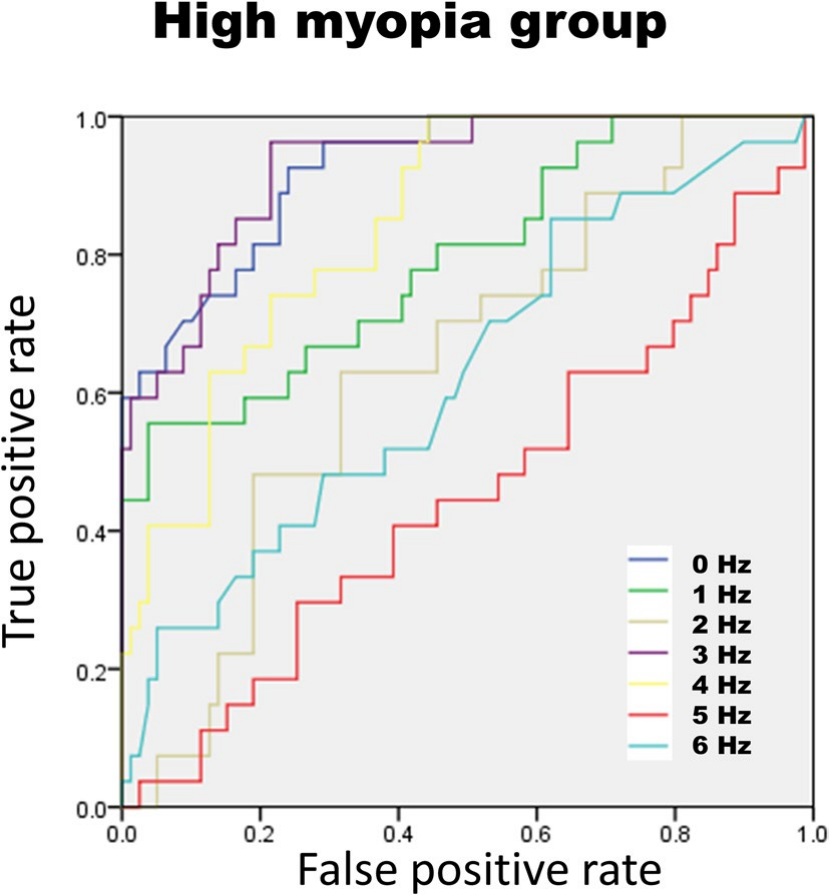
Receiver operating curve of amplitude 0 to 6 Hz frequency for high myopia. In high myopic eyes, the area under curve of 0 Hz decreased and became lower than the area under curve of 3 Hz (0.924 vs 0.927). It is notable that 4 Hz had better area under curve in high myopia than in all subjects. The image was created using SPSS software (ver. 17.0; SPSS Inc, Chicago, IL).

**Figure 3:**
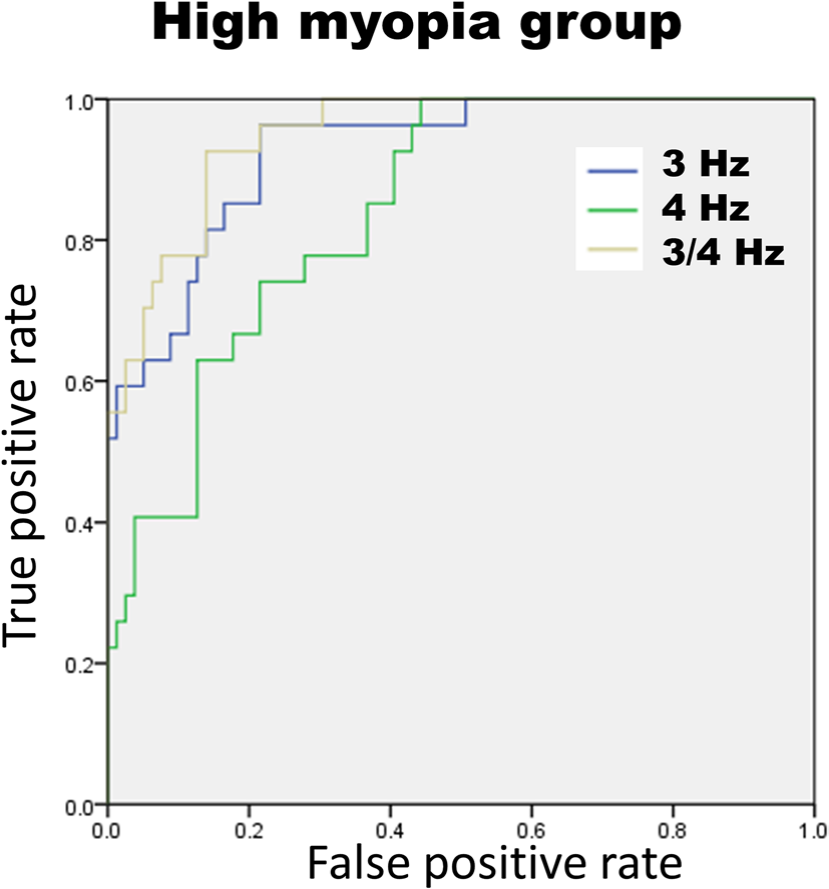
Receiver operating curve of amplitude 3 Hz, 4 Hz, and 3 plus 4 Hz frequency for high myopia. In high myopic eyes, the combination of 3 Hz and 4 Hz had the better area under curve than others. The image was created using SPSS software (ver. 17.0; SPSS Inc, Chicago, IL).

In the high myopic population (n = 106), the average RNFL, each clock-hour RNFL, and the DFT signals were analyzed with the receiver operating curve (ROC) to determine the ability to diagnose glaucoma (Table 5). We found area under the receiver operating curve (AUC) of 0.93 with 95% confident interval (CI) from 0.90 to 0.96 for the amplitude at the 3 Hz frequency (Fig. 1). When the ROC analysis was confined to patients with axial lengths $\ge \hspace{0.17em}26$ mm, the AUCs were 0.93 (95% CI 0.88–0.98) and 0.95 (95% CI 0.91–0.99) at 3 Hz and 3 Hz + 4 Hz frequency, respectively (Figs. 2, 3).

**Table 5:** The area under the receiver operating curve (AUC) of all RNFL parameters and DFT frequency from 0 to 6 Hz in high myopic eyes (N = 106).

Parameters	AUC	95% CI
Cirrus OCT RNFL thickness ($\upmu \mathrm{m})$		
Average RNFL	0.92	0.87–0.98
Clockhour of RNFL		
1	0.61	0.49–0.73
2	0.42	0.30–0.54
3 (nasal)	0.36	0.24–0.48
4	0.4	0.28–0.52
5	0.43	0.30–0.55
6 (inferior)	0.77	0.68–0.86
7	0.94	0.90–0.98
8	0.93	0.89–0.98
9 (temporal)	0.76	0.64–0.87
10	0.87	0.79–0.95
11	0.83	0.75–0.91
12 (superior)	0.57	0.45–0.69
Frequency of DFT		
0 Hz	0.92	0.87–0.98
1 Hz	0.79	0.69–0.90
2 Hz	0.64	0.53–0.76
3 Hz	0.93	0.88–0.98
4 Hz	0.84	0.77–0.92
5 Hz	0.45	0.32–0.58
6 Hz	0.63	0.50–0.75
3 + 4 Hz	0.95	0.91–0.99

*RNFL* retinal nerve fiber layer, *DFT* discrete Fourier transform.

## Discussion

It is difficult to detect glaucomatous RNFL defects in myopic eyes, due to disc torsion, disc tilts, temporal shifts, and vertical asymmetric distributions.^11,12^ In this study, we evaluated the 12-clock-hour RNFL map without defining the start and end points, but as a continuous wave. With this approach, we could eliminate or minimize the effect of disc torsion, or the degree of deviation in the disc-foveal angle. In addition, the other features of myopic eyes could be analyzed with new parameters that described the characteristics of the “wave” of clock-hour RNFL thickness.

To our best knowledge, the biomarker from RNFL in OCT exam to diagnose glaucoma from normal myopic population showed around 0.825–0.826 AUC in previous study^13,14^ (Table 6). The degree of glaucoma seems more light in the present study than in Takuhei et al. and Kim et al. The mean deviation (MD) of VF exam in glaucomatous myopic group was − 8.1 dB in Takuhei’s and − 8.56 dB in Kim’s study. The MD in our glaucomatous myopic group is − 5.40 dB. Our DFT model showed superior to other RNFL based analysis, and could be effective in more early glaucoma.

**Table 6:** The review of Biomarkers of retinal nerve fiber layer to diagnose glaucoma from myopia.

	Country	OCT device	Biomarker	AUROC
Present study	Taiwan	Cirrus SD-OCT	3 Hz	0.927
			4 Hz	0.844
			3/4 Hz	0.949
Takuhei Shoji et al	Japan	RTVue-100	Average RNFL thickness	0.826
Kim NR et al	Korean	RTVue-100	Average RNFL thickness	0.825

*RNFL* retinal nerve fiber layer, *OCT* optical coherence tomography, *AUROC* area under the receiver operator characteristic.

The DFT was used to transform the wave information into a spectrum of amplitudes of different frequencies. In this study, all the information, including all 360° of the 3.46-mm diameter cpRNFL circle recorded with OCT, was set to equal 1 s. Thus, we could gain half number of 12 signal samples in frequency. The output was separated into 7 groups, from 0 to 6 Hz.

The 0 Hz frequency represented the mean of all input signals. Accordingly, a decrease in amplitude from 0 Hz indicated thinning compared to the average RNFL. In the present study, we observed significant decreases associated with aging, OAG, and myopia. Bae et al. pointed out that RNFL thickness decreased in myopic eyes, according to the reading in OCT without adjustment for magnification effect.^15^.

The 1 Hz frequency was defined as one cycle of the wave around 360°° of the RNFL circle. This frequency was associated with increased thickness on one side of the RNFL, and decreased thickness on the opposite side of the RNFL. This pattern may be related to a tilted disc. In our study, the amplitude at 1 Hz was associated with the axial length in the control group. In a previous study, a tilted disc was commonly found in myopic eyes, and it was associated with a decreased RNFL thickness, particularly in myopic eyes with beta-zone parapapillary atrophy.^16^ That finding was consistent with our DFT analysis.

The 2 Hz and 4 Hz frequencies represented two and four cycles around the RNFL circle, respectively. These even-numbered frequencies were associated with increases or decreases in RNFL thicknesses in a symmetrical pattern on opposite sides of the disc. The angle between the peaks of 2 adjacent waves at 2 Hz is 180 degrees, and may be related to the “ISNT” rule of the anatomy of optic disc in human eyes^17^. Typically, the RNFL is thicker in the inferior and superior areas and thinner in the nasal and temporal areas. In the RNFL map of OCT data, this pattern is known as a double-hump configuration^18^. In the present study, the amplitude at 2 Hz was significantly lower in eyes with long axial lengths in the control group. According to a previous histological study, in myopic eyes, the RNFL thickness decreased more in the inferior and superior quadrants than in the nasal and temporal quadrants^17^. Therefore, myopia may appear as a smoother double hump on the RNFL map, and a decrease in the amplitude at 2 Hz in the DFT output. In the 4 Hz spectrum, there are 4 peaks and 4 troughs around one circle, thus, each 2 adjacent peaks are perpendicular. In the control group, greater amplitudes were observed at 4 Hz in subjects with longer axial lengths. This pattern may be associated with a “temporal shift” effect in the double hump configuration in the RNFL map of OCT data.

The 3 Hz frequency represents the characteristic of angle between 2 peaks about 120 degrees, such as the angle between temporal retinal arcades. It is vulnerable to asymmetric early glaucomatous NFL change, which causes the uneven flatten of the double hump. The biomarker will decrease even in more severe glaucomatous eye, because the decreased height of double hump would be associated with decreased amplitude at 3 Hz frequency. Regarding the effect of myopic change in the eye, axial length elongation, tilt disc, and temporal shift of retinal arcade impact many biomarkers which were used to evaluate glaucoma. In the present study, we found that the amplitude at 3 Hz frequency was invulnerable to axial length elongation (Table 2). On the other hand, 3 Hz frequency is relatively invulnerable to tilt disc and general NFL thinning. Because the general decrease in NFL would not obliterate the current characteristic of 3 Hz. However, since we assumed that the temporal retinal arcade plays an important role in the amplitude at 3 Hz frequency, the temporal shift effect of myopic change may cause less component at 3 Hz frequency but more at 4 Hz frequency at the same time. The characteristic of amplitude at 3 Hz frequency might be extraordinary, and 4 Hz frequency would be the adjunctive candidate biomarker in high myopic eyes.

The amplitudes at 3 Hz was considered as the candidate biomarker with ROC AUCs of 0.93 (95% CI 0.90–0.96) and 0.93 (95% CI 0.88–0.98) in all subjects and high myopic eyes, respectively. When the 3 Hz amplitude was less than 70 μm, it showed a specificity of 0.89 and sensitivity of 0.78 for diagnosing glaucoma in high myopic eyes. On the other hand, the 4 Hz amplitude had the characteristic of increase in myopia and decrease in glaucomatous eyes. It could also have the power to differentiate glaucoma from high myopia. In the ROC analysis of diagnosing glaucoma in high myopia, setting mean of amplitude at 3 Hz and 4 Hz as new parameter would have AUC of 0.95 (95% CI 0.91–0.99) and achieve 0.89 in sensitivity and 0.86 in specificity.

It is interesting that we found that the 4 Hz amplitude was an outstanding adjunctive biomarker in high myopic eyes. Because the temporal shift of the RNFL was observed in patients with high myopia, we hypothesized that the angle between the superior and inferior bundles in the nerve fiber layer decreased from 120° to approximate 90°, in addition to the elongation of the axial length.^12^ Some components of the wave of the 3 Hz frequency might have shifted to the 4 Hz frequency.

The original OCT RNFL map has been a great tool for detecting and monitoring glaucoma and the early progression of the disease.^19^ However, to the best of our knowledge, the signs of glaucoma of this anatomy-based analysis could be confused with myopic changes, such as peripapillary atrophy, disc torsion, and tilt.^20^ There may be different patterns of RNFL change for different types of pathogenesis, which might not be differentiated, based only on a focal loss of the RNFL detected with OCT. In contrast, with the DFT analysis, we could detect the characteristics of the defect from other information. For example, there might be various amounts of compensation in the whole nerve fiber layer, according to different types of pathogenesis, even though these various conditions might all result in a similar focal nerve fiber defect. The advantage of DFT analysis is that it can differentiate among different conditions by considering the entire 360° area of the RNFL at the same time. Moreover, the DFT is simple and convenient to perform; it only requires entering 12 numbers into the function, and it does not require massive computing time.

This study had some limitations. First, it was a cross-sectional study and lacked information for longitudinal analysis. Further study is needed to support and extend the results. Second, the RNFL clock-hour map provided discrete data; therefore, we chose the discrete Fourier transform for the analysis. Thus, in the DFT analysis, we could only assess up to 6 Hz when analyzing the 12 equidistant points. More equidistant points would have enabled a more precise interpretation, including information at higher frequencies. However, most of the major characteristics were found in the lower parts of the frequency domain. Third, although we assessed the entire 360° of the RNFL, we could not rule out the possibility that there might be other, unknown sources of pathogenesis that might contribute to RNFL damage, and this could have affected the results. Therefore, further study is needed to eliminate that possibility or discover the other factors.

In conclusion, we demonstrated a new model for a DFT analysis of RNFL with Cirrus HD-OCT. It is a useful biomarker for diagnosing glaucoma in high myopic eyes, which is difficult by existing method. Analyses of amplitudes in different frequencies is a potential diagnostic tool for further clinical practice.

## Methods

This study included 232 eyes from 232 patients who were retrospectively collected in the Glaucoma Department of the Taipei Veteran General Hospital, from September 2016 to February 2017, adhered to the tenets of the Declaration of Helsinki. Institutional Review Board (IRB)/Ethics Committee approval was obtained from Taipei Veterans General Hospital, Taiwan. The informed consent was obtained from all subjects. All eyes received comprehensive measurements, including auto-refraction, intraocular pressure (IOP), axial length (measured with an IOLMaster, V.5.02, Carl Zeiss Meditec, Oberkochen, Germany), slitlamp biomicroscopy, gonioscopy, fundus photography, and visual field evaluations. All subjects underwent pupil dilation for imaging with the Cirrus HD-OCT (Carl Zeiss, Meditec), which was always operated by the same technician. In the Cirrus HD-OCT, the optic disc was automatically centered when measuring the cpRNFL; images that were not properly centered in the test outcome were re-mapped manually by one of the investigators (YFC) to ensure accurate centration. The retinal thicknesses at points along the 12-clock-hour sector cpRNFL map were recorded for analysis. Then, this information was input into the computer as a discrete function. A visual field (VF) test was performed with Humphrey automated perimetry, supplemented with the Swedish Interactive thresholding algorithm (SITA) standard (i750, Carl Zeiss Meditec, Oberkochen, Germany). VF tests were regarded reliable, when the fixation loss was ≤ 25%, false positives were ≤ 15%, and false negatives were ≤ 15%.

### Control group and glaucomatous group

All participants had a best-corrected visual acuity no worse than 20/30, an IOP < 21 mmHg with or without glaucoma medication, astigmatism ≤ 3 D, and reliable SITA-Standard 24-2 VF test results, within 4 months of OCT imaging. In the control group, all participants had normal IOPs (< 21 mmHg), unremarkable anterior segment and fundus findings, except for a tilted disc and peripapillary chorioreinal atrophy which did not interfere with cpRNFL evaluations with the SD-OCT. We did not exclude subjects with general reductions in VF sensitivity or enlarged blind spots which corresponded to high myopic fundus changes. When both eyes met the enrollment criteria, we chose the right eye for statistical analysis. High myopia was defined as axial length $\ge 26\mathrm{m}\mathrm{m}.$ Glaucoma was diagnosed based on characteristic changes in optic nerve head (ONH)/RNFL and corresponding VF loss. The ONH changes included neuroretinal rim thinning, notching, or excavation. A glaucomatous RNFL defect on fundus photography was slit-, wedge-, or band-shaped, matching RNFL distribution pattern and were wider than adjacent vessels. Glaucomatous VF defect was defined as follows: (1) conformed to patterns of retinal nerve fiber distribution; (2) had ≥ 3 non-edge continuous points in the same with a pattern standard deviation value < 0.05 and one < 0.01; and (3) with a glaucoma hemifield test classified as outside normal limits. Participants will have at least three meaningful glaucomatous visual field defects. When both eyes of one subject fulfilled all the criteria, we chose the eye with better VF for statistical analysis.

We excluded eyes with coexisting ocular disease (other than a refractive error or cataract); ocular inflammation; a history of ocular surgery within 3 months; or a history of refractive surgery or ocular trauma.

### Discrete Fourier transform (DFT)

**Figure 4:**
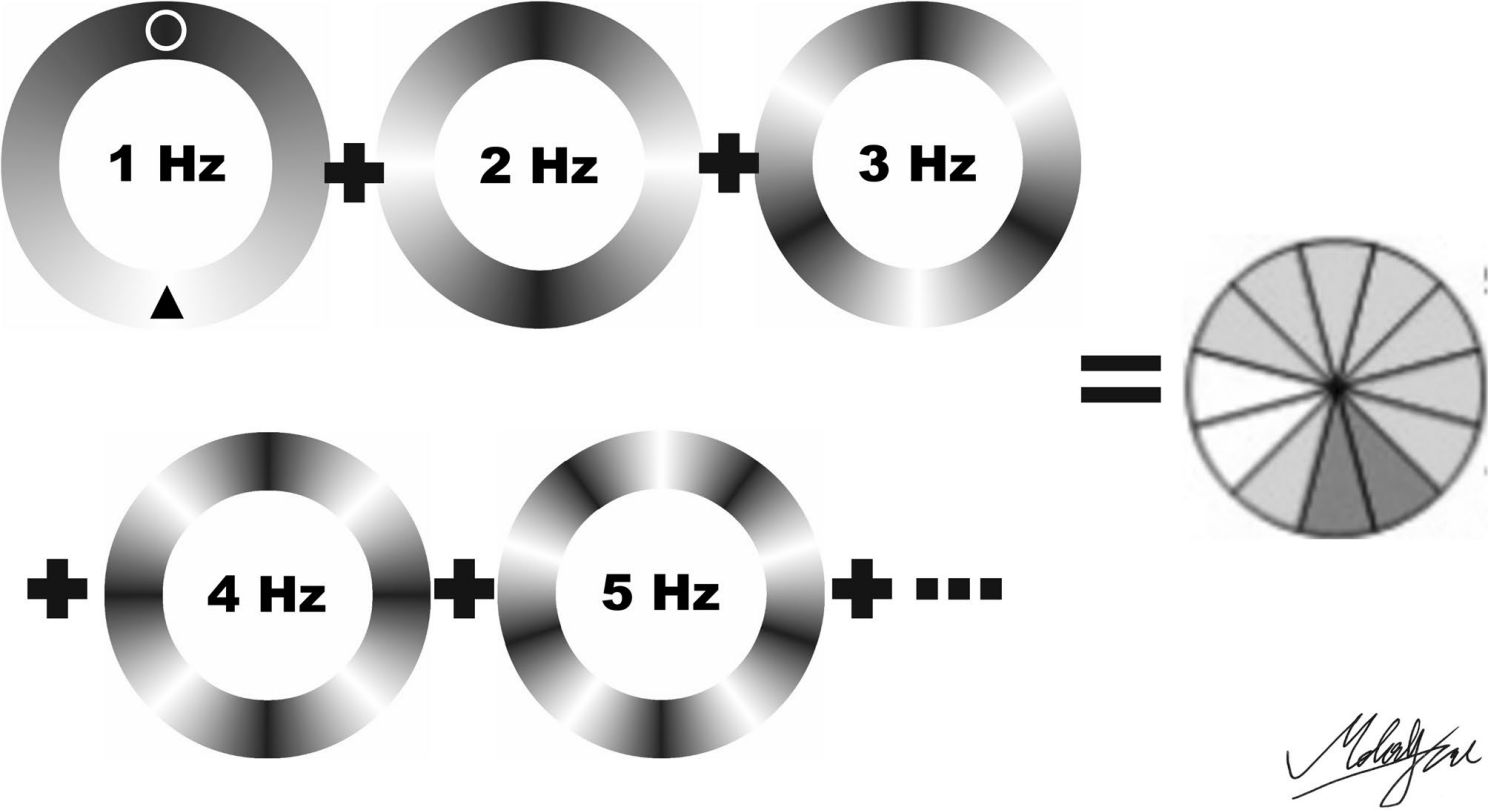
Sketch of discrete Fourier transform. 12 equidistant data of thickness of circumpapillary retina nerve fiber layer could be represented as the combination of consecutive different frequency of the thickness. In 1 Hz, for example, the circle symbol indicates the peak, and the triangle symbol indicates the trough, so there is one cycle in a round (1 Hz). The image was created using Adobe Illustrator software (ver. Creative Cloud 2018; Adobe Inc. San Jose, CA).

For the DFT,^21^ we input 12 samples, the number of measurements at sites around the 12-clock-hour RNFL map (Fig. 4). We implemented the following settings: sampling time = 1/12 s, frequency = 1 Hz, and delta frequency = 1 Hz. The DFT formula, with N = 12, was calculated as follows:

1 $${X}_{xn}=\sum_{k=0}^{N-1}{(X}_{k})({e}^{2\pi ikn/N})$$

The outputs included the amplitude and the initial phase of the wave for each frequency. For more detail, please see the Ref.^21^. In this study, we analyzed and interpreted the amplitude at each frequency. All calculations were performed with Microsoft Excel 2010 (Microsoft, Redmond, Washington, US) and Visual Basic for Applications. Figure 4 DFT conducted by the input-12 numbers of 12-clock-hour map from OCT.

### Statistical analysis

Statistical analyses were performed with commercially available software (SPSS ver. 17.0; SPSS Inc, Chicago, IL). The t-test and ANOVA were used to analyze differences in RNFL thickness and frequency between groups. A generalized linear model was used to identify the effects of myopia and glaucoma on each parameter. A receiver operating characteristic (ROC) curve was used to analyze the sensitivity and specificity of potential predictive factors. *P* < 0.05 was considered statistically significant.
